# Use of Direct LAMP Screening of Broiler Fecal Samples for *Campylobacter jejuni* and *Campylobacter coli* in the Positive Flock Identification Strategy

**DOI:** 10.3389/fmicb.2016.01582

**Published:** 2016-09-30

**Authors:** Islam I. Sabike, Ryoko Uemura, Yumi Kirino, Hirohisa Mekata, Satoshi Sekiguchi, Tamaki Okabayashi, Yoshitaka Goto, Wataru Yamazaki

**Affiliations:** ^1^Department of Veterinary Sciences, Faculty of Agriculture, University of MiyazakiMiyazaki, Japan; ^2^Department of Food Control, Faculty of Veterinary Medicine, Benha UniversityBenha, Egypt; ^3^Center for Animal Disease Control, University of MiyazakiMiyazaki, Japan; ^4^Organization for Promotion of Tenure Track, University of MiyazakiMiyazaki, Japan

**Keywords:** foodborne, *Campylobacter jejuni*, *Campylobacter coli*, feces, chickens, separation strategy, LAMP

## Abstract

Rapid identification of *Campylobacter*-positive flocks before slaughter, following freezing and heat treatment for the *Campylobacter*-positive carcasses at the slaughterhouses is an effective control strategy against foodborne campylobacteriosis. We evaluated a loop-mediated isothermal amplification (LAMP) assay for the direct screening of naturally contaminated chicken cloacal swabs for *C. jejuni*/*C. coli* to compare this assay with conventional quantitative culture methods. In a comparison study of 165 broilers, the LAMP assay showed 82.8% (48/58 by conventional culture) sensitivity, 100% (107/107) specificity, 100% (48/48) positive predictive value (PPV), and 91.5% (107/117) negative predictive value (NPV). In a comparison of 55 flocks, LAMP showed 90.5% (19/21) sensitivity, 100% (34/34) specificity, 100% (19/19) PPV, and 94.4% (34/36) NPV. In the cumulative total of 28 farm-level comparisons, LAMP showed 100% (12/12) sensitivity, 100% (16/16) specificity, 100% (12/12) PPV, and 100% (16/16) NPV. The LAMP assay required less than 90 min from the arrival of the fecal samples to final results in the laboratory. This suggests that the LAMP assay will facilitate the identification of *C. jejuni*/*C. coli*-positive broiler flocks at the farm level or in slaughterhouses before slaughtering, which would make it an effective tool in preventing the spread of *Campylobacter* contamination.

## Introduction

Campylobacteriosis is among the most frequently reported foodborne diseases worldwide. While numerous potential vehicles of transmission exist, commercial chicken meat has been identified as one of the most important food vehicles for this organism (World Health Organization [WHO], 2010). The disease burden of campylobacteriosis and its sequelae is 0.35 million disability-adjusted life years (DALYs) per year and total annual costs are €2.4 billion (European Food Safety Authority [EFSA], 2011). In 2011, foodborne disease caused by *Campylobacter* spp. led to an estimated loss of 6099 DALYs in Japan (Kumagai et al., 2015). The prevalence of *Campylobacter* in broiler flocks in Japan was 47.2% in 2009–2010 and 44.0% in 2013–2014 (Haruna et al., 2012; Yamazaki et al., 2016). The primary source of contamination of the broiler production chain occurs at the farm (Johannessen et al., 2007). Because in naturally contaminated broiler flocks, after the first birds in a flock become colonized by the pathogen, fecal shedding of 6.00 log *Campylobacter* cells per gram of feces, combined with coprophagy, leads to the rapid transmission of infection throughout the flock (Wagenaar et al., 2006, 2008). Moreover, the intestinal tract of chickens, especially the ceca and large intestine, can harbor a large number of *Campylobacter* species. (Stern et al., 1995; Berrang et al., 2001). Control of *Campylobacter* in primary broiler production should provide greater public health benefits than control later in the chain as the bacteria may also spread from farms to humans by other pathways than broiler meat (European Food Safety Authority [EFSA], 2011). One of the primary uses of rapid methods is fast screening of a large number of samples. Among these newer rapid techniques, one promising candidate is loop-mediated isothermal amplification (LAMP; Notomi et al., 2000). In previous studies, LAMP assays were successfully used to rapidly detect *C. jejuni* and *C. coli* in naturally contaminated chicken meat and human stool samples (Yamazaki et al., 2008, 2009; Pham et al., 2016). Here, we assessed the feasibility of using LAMP in the scheduled slaughtering strategy for identifying positive flocks before slaughter at the farm level. We performed direct LAMP screening of *C. jejuni* and *C. coli* in 165 broiler fecal samples.

## Materials and Methods

### Bacterial Strains

*Campylobacter* reference strains were obtained from the Japan Collection of Microorganisms (JCM). *C. jejuni* subsp. *jejuni* JCM2013 = ATCC29428 and *C. coli* JCM2529^T^ = ATCC33559^T^ are strains isolated from the diarrheic stool of a child and a pig, respectively, which are capable of producing human campylobacteriosis. The two reference strains were used for quality control and determination of analytical sensitivity of the LAMP assay in artificially spiked broiler fecal samples.

### Isolation of *C. jejuni* and *C. coli* from Chicken Broiler Fecal Samples

A total of 165 chicken broiler fecal samples (cloacal swabs) were collected from 41 flocks at 8 of a total of 28 broiler farms in Kyushu, Japan, between June 2015 to February 2016. Of the 41 flocks, 14 were sampled twice at approximately week 4 (days 26–35) and week 6 (days 39–48). The remaining 27 flocks were sampled once at approximately week 6 (days 39–48). The mean age of tested broilers was 41 days, (range, 26–48 days). Each sampling was carried out for two flocks at two different broiler houses in one firm, except for one sampling (X2, September 2015, Supplementary Table 1). Three live broilers from each flock were randomly chosen in the broiler house, from which fecal samples were retrieved directly from the cloaca using sterilized cotton swabs and then aseptically collected into sterilized 50 mL polypropylene tubes. All samples were kept at 4°C, and then cultured within 24 h of arrival, except for samples obtained on Friday afternoons, which were cultured within 72 h of arrival. Details of the samples are shown in Supplementary Table 1.

*Campylobacter* cells were isolated from fecal samples both by direct plating for quantitative isolation and by selective enrichment followed by plating for qualitative isolation. For direct plating, serial 10-fold dilutions of the fecal samples in phosphate-buffered saline (PBS, pH 7.2) were prepared. 100 μL of each fecal dilution between 10^-2^ to 10^-6^ was inoculated onto modified charcoal cefoperazone deoxycholate agars (mCCDA, Oxoid; Hampshire, UK) with a sterile disposable bacteria spreader (Heathrow Scientific; Vernon Hills, IL, USA), and then incubated at 42°C for 44–48 h under microaerobic atmosphere conditions (approximately 8% O_2_, 7% CO_2_, and 85% N_2_). After the 10% homogenate preparation in PBS (pH 7.2), fecal samples were kept at -80°C until DNA extraction. Thereafter, the leftovers of the fecal samples after preparation of 10-fold serial dilutions were inoculated into tubes containing 9 mL of Preston enrichment broth (Oxoid) supplemented with 5% (v/v) lysed horse blood, and thoroughly mixed with a Vortex-Genie 2 (Scientific Industries Inc.; NY, USA). The samples were then incubated at 42°C for 20–24 h under microaerobic conditions. For isolation of *C. jejuni* and *C. coli*, a loopful (approximately 10 μL) of Preston enrichment broth culture was stroked onto Butzler (Oxoid) and mCCDA media, and then incubated at 42°C for 44–48 h under microaerobic conditions. A maximum of five typical *Campylobacter*-like colonies were chosen from the mCCDA and/or Butzler agars. Matrix-assisted laser desorption ionization (MALDI) Biotyper-MA2 (Bruker Daltonics Inc., Billerica, MA, USA) was used to identify either *C. jejuni* or *C. coli*, in accordance with the manufacturer’s instructions.

### LAMP Assay

To enhance the detection sensitivity of the LAMP assay, we used the protocol of a three-step centrifugation procedure for DNA extraction developed in our previous study (Yamazaki et al., 2009). The LAMP assay targeting two sequences, a presumed oxidoreductase gene in *C. jejuni* and a *gufA* gene in *C. coli*, was carried out as previously described (Yamazaki et al., 2008, 2009; Yamazaki, 2013). The sensitivity of the LAMP assay was determined using chicken fecal samples spiked with *C. jejuni* JCM 2013 and *C. coli* JCM 2529^T^, according to the previous studies (Yamazaki et al., 2009; Yamazaki, 2013).

### Statistical Analysis

Prevalence estimates for *Campylobacter* and LAMP performance were analyzed using Fisher’s exact two-tailed test analyses with R ver. 3.1.3 (R Foundation for Statistical Computing, Vienna, Austria), with differences considered significant with *P* < 0.05, as well as using analyses of sensitivity, specificity, positive and negative predictive values, ROC and AUC with Microsoft Excel 2016 (Microsoft Excel Ver. 3. 2013., Microsoft Corp., Redmond, WA, USA) and SPSS Version 20 (IBM Corp. Released 2011. IBM SPSS Statistics for Windows, Version 20.0. IBM, Armonk, NY, USA).

## Results and Discussion

The feasibility of using direct LAMP screening as part of the scheduled slaughtering strategy for identifying positive flocks before slaughter was carried out with 165 broiler fecal samples. The assay enabled simple and rapid detection less than 90 min from the beginning of DNA extraction to final assessment. Furthermore, very good to excellent diagnostic performance was obtained with high AUCs of 0.912, 0.952 and 1.0 in 165 individual broiler fecal samples, 55 flocks, and 29 cumulative farms by the assessment with receiver operating characteristic (ROC) curves. **Tables 1** and **2** show the effectiveness of the direct screening method in the detection of *C. jejuni* and *C. coli* in broilers.

**Table 1:** Comparison of *Campylobacter jejuni*/*Campylobacter coli* isolation by conventional culture methods and LAMP screening results in 165 broiler fecal samples.

	LAMP-positive (*n* = 48)		LAMP-negative (*n* = 117)
	*C. jejuni*	*C. coli*	
**Culture results**			
*C. jejuni*-positive (*n* = 50)	41	0	9
*C. coli*-positive (*n* = 6)	2	5	1
*C. jejuni*/*C. coli*-positive (*n* = 2)	2	2	0
*C. jejuni*/*C. coli*-negative (*n* = 107)	0	0	107

**Table 2:** Comparison of *C. jejuni*/*C. coli* population and LAMP screening results in 58 culture-positive broiler fecal samples.

Culture results		*C. jejuni*/*C. coli* LAMP		Concordance of culture and LAMP results (%)
*C. jejuni*/*C. coli* enumeration (log cfu/g)	No. of culture-positive (*n* = 58)	No. of LAMP-positive (*n* = 48)	No. of LAMP- negative (*n* = 10)	
≥9.00	1	1	0	100%
≥8.00 - <9.00	2	2	0	100%
≥7.00 - <8.00	13	13	0	100%
≥6.00 - <7.00	15	15	0	100%
≥5.00 - <6.00	9	8	1	88.9%
≥4.00 - <5.00	8	4	4	50.0%
≥3.00 - <4.00	2	0	2	0%
<3.00	8^∗^	5	3	62.5%

*^∗^Of the eight samples, all tested negative by direct plating, but five tested positives with enrichment culturing.*

The results of LAMP assay were compared statistically with both the quantitative and qualitative results generated by the conventional culture methods for the isolation of *C. jejuni* and *C. coli.* As shown in **Table 1** and Supplementary Table 1, when conventional culture methods were used, *C. jejuni* and/or *C. coli* was isolated from 58 of the 165 chicken fecal samples, of which 50 chicken fecal samples were *C. jejuni*-positive, six were *C. coli*-positive, and two were positive for both. When the LAMP assay was performed, 48 of the 165 chicken fecal samples were *C. jejuni-*and/or *C. coli*-positive. The 48 *C. jejuni*/*C. coli*-positive LAMP chicken fecal samples were all positive by culture methods, but 10 of 58 samples that were *C. jejuni*/*C. coli*-positive by conventional culture methods tested negative with the LAMP assay i.e., false-negative. To clarify, the LAMP method applied in this study detected 48 *C. jejuni*/*C. coli-*positive chicken fecal samples, composed of 40 *C. jejuni*-positive, two *C. coli*-positive, and six both *C. jejuni-* and *C. coli*-positive samples (**Table 1**). Among the ten false-negative fecal samples by LAMP, nine samples were identified as *C. jejuni-*positive and one as *C. coli-*positive by the culture method. The LAMP assay was true-negative for all 107 *C. jejuni*/*C. coli* culture-negative fecal samples. The results of statistical analysis were as follows: 82.8% (48 of 58 samples) sensitivity, 100% (107/107) specificity, 100% (48/48) PPV, and 91.5% (107/117) NPV. For accurate evaluation, the discriminant performance of the LAMP assay was measured using ROC curves using the conventional culture methods as the objective standard. The ROC generated an AUC of 0.914 with a significance level of (*P* < 0.001) for the LAMP assay for detecting *C. jejuni* and/or *C. coli* in chicken fecal samples in comparison with the culture methods. In the 55 individual flock level comparison, the results of the LAMP assay generated 90.5% (19/21) sensitivity, 100% (34/34) specificity, 100% (19/19) PPV and 94.4% (34/36) NPV. Furthermore, in the 28 farm level comparison, the results of the LAMP assay generated 100% (12/12) sensitivity, 100% (16/16) specificity, 100% (12/12) PPV and 100% (16/16). The LAMP assay was estimated to have an AUC of 0.952 and 1.0 in flock and farm level analyses, respectively. The sensitivities of the LAMP assay for *C. jejuni* and *C. coli* in artificially spiked fecal samples were 3.89 and 3.60 log cfu/g of fecal sample. Quantitative analysis of *C. jejuni*/*C. coli* by conventional culture methods resulted in a wide range from <3.00 to 9.00 log cfu/g of feces (**Table 2** and Supplementary Table 1).

While no false-positives were observed in the three analysis levels, false-negatives were confirmed in individual (17.2%, 10/58) and cumulative flock (10.5%, 2/21) levels, but were perfectly matched in cumulative farm (0%, 0/12) level analysis. This outcome demonstrates that the LAMP assay has sufficient potential to identify *C. jejuni*/*C. coli*-posiive flocks/farms by direct detection of *C. jejuni*/*C. coli* from at least one aliquot of naturally contaminated broiler feces in a small number of samples (three to six), despite the influence of both inhibitors and small amounts of *C. jejuni*/*C. coli* cells in broiler fecal samples. As shown in Supplementary Table 1, among the 17.2% (10/58) LAMP false-negatives, five were observed in the six chosen broilers for flocks U4A and U4B in the same farm at the same sampling, which means that LAMP false-negative frequency was 83.3% (5/6) for *C. jejuni*/*C. coli* ranging from <3.00 to 5.81 log cfu/g in farm U in September, 2015. In the six chosen broilers, one of six samples showed both culture and LAMP positivity (No. 40, U4B–5A). In contrast, all six culture-positive samples (Nos. 64–66 in flock U6A and nos.67–69, in flock U6B; Supplementary Table 1), ranging from 6.56 to 9.00 log cfu/g of fecal samples, showed positive for *C. jejuni*, which were chosen from two flocks in the same farm U in November, 2015. Therefore, the false-negative LAMP results may have been caused by inhibitors of DNA amplification, possibly derived from common feeding, as well as by low *C. jejuni*/*C. coli* cell numbers in the fecal samples from farm U in September 2015. The remaining five LAMP false-negatives were rare phenomena in each sampling, observed with a lower LAMP false-negative rate, ranging from 16.6% (1/6) to 33.3% (1/3). Although 17.2% (10/58) of false-negatives were observed in the individual cumulative flock assay using LAMP, high concordance at 90.5% (19/21) of diagnostic sensitivity and at 100% (34/34) of diagnostic sensitivity were obtained in the cumulative flock level analysis, as well as perfect concordance at 100% (12/12) of diagnostic sensitivity and at 100% (16/16) of diagnostic sensitivity in the cumulative farm level analyses.

This discrepancy in false-negative LAMP results may be due to the small number of *C. jejuni*/*C. coli* and/or inhibitory factors in the chicken fecal samples. To investigate the effect of the second factor, the supernatants extracted by the three centrifugation steps of the ten *C. jejuni*/*C. coli* LAMP false-negative samples were diluted between 2- to 25-fold and retested by LAMP. These assays were all negative. An effect of fecal inhibitors on DNA amplification in chicken fecal samples tested for real-time PCR detection of thermophilic campylobacters has been identified and studied (Lund et al., 2004). In that study, a common problem of real-time PCR was failure of DNA amplification due to inhibitors in the fecal samples, which decreased or completely prevented amplification, producing false-negative results (Wilson, 1997; Lund et al., 2004). Of the 58 *C. jejuni*/*C. coli* culture-positive samples, 38 samples containing *C. jejuni*/*C. coli* at a minimum of 5.00 log cfu/g of feces were positive in LAMP screening. One sample (No. 38, U4A–5B) at 5.81 log cfu/g (**Table 2**; Supplementary Table 1) tested false-negative. LAMP false-negative results were also observed in two flocks in one farm at the same sampling period (Nos. 37–39 in flock U 4A and Nos. 41–42, in flock U4B; Supplementary Table 1), possibly caused by the low *C. jejuni*/*C. coli* loads in fecal samples, which ranged from <3.00–5.81 log cfu/g, as well as inhibitors in the fecal components (Wilson, 1997; Yamazaki et al., 2008).

Some broiler fecal samples caused relatively high growth of background flora onto mCCDA agar in direct plating, ranging from 3.00–6.00 log cfu/g of feces level, causing false-negative results. Supplemental use of a combination of Preston enrichment broth culture and subculturing onto two selective media, mCCDA and Butzler agars, successfully recovered *C. jejuni*/*C. coli*, despite the false-negative results obtained with direct plating (**Table 2**). This explains why four of five direct plating-negative but enrichment culture-positive samples in the same sampling period and farm were positive with LAMP between 24 and 41 min. (No. 52-55, farm S in October 2015, Supplementary Table 1). In fact, the six samples from farm S in October 2015 showed higher background flora, ranging from 5.00–6.00 log cfu/g, which made it difficult to enumerate *Campylobacter* colonies on the mCCDA agars. Three other direct-plating-negative but enrichment-culture-positive fecal samples presumably contained low numbers of *C. jejuni*/*C. coli* cells, because other fecal samples from same flock showed <3.00–5.81 cfu /g of *C. jejuni*/*C. coli* cells (No. 28, No. 39 and No. 45; Supplementary Table 1). In real-time PCR reactions using spiked chicken fecal samples, fecal components significantly decreased the DNA amplification efficacy of samples containing a large number of *C. jejuni*; moreover, positive signals disappeared in samples containing a small number of *C. jejuni* (Lund et al., 2004). It is therefore understandable that the three fecal samples were LAMP-negative, due to the combination of low *C. jejuni*/*C. coli* cell numbers and DNA amplification inhibitors in the fecal components. Identification and remediation of these inhibitors is vital.

The effectiveness of logistic approach to slaughter by processing negative flocks before positive flocks to prevent the spread of the chicken meat contamination has been shown to be an ineffective approach for *Campylobacter* intervention, since it was shown that cross contamination by *Campylobacter* between flocks at the poultry processing facilities was limited (Johannessen et al., 2007). One of the most promising interventions for reducing the spread of contamination in the processing facility is scheduled slaughtering for identifying positive flocks before slaughter, following special treatment for their carcasses such as freezing and heat treatment (Havelaar et al., 2007; World Health Organization [WHO], 2010; European Food Safety Authority [EFSA], 2011). The LAMP assay described here demonstrates the potential for application to a *C. jejuni*/*C. coli-* positive flock identification strategy in farm or processing facilities. Subsequently enhance and facilitate scheduled slaughtering approach to slaughter by subjecting carcasses from positive flocks to treatments or interventions such as freezing, heat, and chemical treatments, which can reduce *Campylobacter* counts. Further work is now required to minimize false LAMP-negative results, which would enable more accurate detection without large numbers of samples, and time-consuming and expensive DNA extraction kits. Deployment to pen-side diagnosis using a portable LAMP device could expand the usability of our current study (Howson et al., 2015). This LAMP assay might be a candidate for the successful identification of *C. jejuni*/*C. coli*-positive flocks to reduce contamination risk in processing facilities, and thereby contribute to the prevention of both the spread of *C. jejuni*/*C. coli* contamination to chicken meat products and of human food poisoning caused by the two bacteria.

## Author Contributions

IS and WY carried out LAMP. RU, YK, HM, SS, TO, and YG carried out sampling and bacteriological examination. WY coordinated the research.

## Conflict of Interest Statement

The authors declare that the research was conducted in the absence of any commercial or financial relationships that could be construed as a potential conflict of interest.
