# Supplementary material for: Use of Direct LAMP Screening of Broiler Fecal Samples for Campylobacter jejuni and Campylobacter coli in the Positive Flock Identification Strategy
Source: Front Microbiol. 2016 Sep 30;7:1582. doi: 10.3389/fmicb.2016.01582 (PMC5043150; doi:10.3389/fmicb.2016.01582)
Supplement: Supplementary file 1 [file Data_Sheet_1.pdf]

Supplemental Table 1.

Results of LAMP time and conventional culture methods for the detection, isolation and count of *C. jejuni* and *C. coli* in chicken broiler fecal samples.

Serial NO.	Sample ID	Date of sampling	Age of chicken	LAMP time		*Campylobacter* count (log CFU/g)	Isolation	
				C. jejuni	*C. coli*		*C. jejuni*	*C. coli*
1	M2A-5A	2015/07/01	44	-	-	-	-	-
2	M2A-5B	2015/07/01	44	-	-	-	-	-
3	M2A-5C	2015/07/01	44	-	-	-	-	-
4	M2B-5A	2015/07/01	44	-	-	-	-	-
5	M2B-5B	2015/07/01	44	-	-	-	-	-
6	M2B-5C	2015/07/01	44	-	-	-	-	-
7	U2A-5A	2015/07/07	44	-	-	-	-	-
8	U2A-5B	2015/07/07	44	-	-	-	-	-
9	U2A-5C	2015/07/07	44	-	-	-	-	-
10	U2B-5A	2015/07/07	44	-	-	-	-	-
11	U2B-5B	2015/07/07	44	-	-	-	-	-
12	U2B-5C	2015/07/07	44	-	-	-	-	-
13	Y2A-5A	2015/07/08	45	25:36	-	6.40	+ve	-
14	Y2A-5B	2015/07/08	45	25:24	-	4.32	+ve	-
15	Y2A-5C	2015/07/08	45	29:30	-	6.53	+ve	-
16	Y2B-5A	2015/07/08	45	22:00	-	5.53	+ve	-
17	Y2B-5B	2015/07/08	45	20:18	-	7.15	+ve	-
18	Y2B-5C	2015/07/08	45	-	-	4.73	+ve	-
19	S2A-5A	2015/07/29	46	25:48	37:36	5.53	-	+ve
20	S2A-5B	2015/07/29	46	29:18	-	4.23	+ve	-
21	S2A-5C	2015/07/29	46	36:06	40:30	4.61	+ve	+ve
22	S2B-5A	2015/07/29	46	27:30	51:12	4.20	+ve	-
23	S2B-5B	2015/07/29	46	32:12	33:24	5.49	-	+ve
24	S2B-5C	2015/07/29	46	22:00	-	6.60	-	+ve
25	Z2A-5A	2015/08/27	44	36:24	-	7.60	+ve	-
26	Z2A-5B	2015/08/27	44	34:00	-	6.80	+ve	-
27	Z2A-5C	2015/08/27	44	34:54	-	7.62	+ve	-
28	Z2B-5A	2015/08/27	44	-	-	< 3.00 *	+ve	-
29	Z2B-5B	2015/08/27	44	-	-	-	-	-
30	Z2B-5C	2015/08/27	44	-	-	-	-	-
31	M4A-5A	2015/09/04	43	-	-	-	-	-
32	M4A-5B	2015/09/04	43	-	-	-	-	-
33	M4A-5C	2015/09/04	43	-	-	-	-	-
34	M4B-5A	2015/09/04	43	-	-	-	-	-
35	M4B-5B	2015/09/04	43	-	-	-	-	-
36	M4B-5C	2015/09/04	43	-	-	-	-	-
37	U4A-5A	2015/09/07	42	-	-	4.45	+ve	-
38	U4A-5B	2015/09/07	42	-	-	5.81	+ve	-
39	U4A-5C	2015/09/07	42	-	-	< 3.00 *	+ve	-
40	U4B-5A	2015/09/07	42	43:48	-	5.23	+ve	-

41	U4B-5B	2015/09/07	42	-	-	4.28	+ve	-
42	U4B-5C	2015/09/07	42	-	-	3.90	+ve	-
43	X2-5A	2015/09/07	40	-	-	3.70	-	+ve
44	X2-5B	2015/09/07	40	-	56:24	5.04	-	+ve
45	X2-5C	2015/09/07	40	-	53:12	< 3.00 *	-	+ve
46	T2A-5A	2015/09/24	45	44:24	-	7.30	+ve	-
47	T2A-5B	2015/09/24	45	25:00	16:24	6.57	+ve	-
48	T2A-5C	2015/09/24	45	37:48	14:00	6.96	+ve	+ve
49	T2B-5A	2015/09/24	45	24:00	-	7.63	+ve	-
50	T2B-5B	2015/09/24	45	20:42	-	8.67	+ve	-
51	T2B-5C	2015/09/24	45	27:00	-	6.40	+ve	-
52	S4A-5A	2015/10/05	44	24:12	-	< 3.00 *	+ve	-
53	S4A-5B	2015/10/05	44	40:18	-	< 3.00 *	+ve	-
54	S4A-5C	2015/10/05	44	34:24	-	< 3.00 *	+ve	-
55	S4B-5A	2015/10/05	44	34:24	-	< 3.00 *	+ve	-
56	S4B-5B	2015/10/05	44	-	-	< 3.00 *	+ve	-
57	S4B-5C	2015/10/05	44	33:48	-	6.20	+ve	-
58	Z4A-5A	2015/10/22	41	32:24	-	6.98	+ve	-
59	Z4A-5B	2015/10/22	41	38:24	-	6.45	+ve	-
60	Z4A-5C	2015/10/22	41	-	-	4.78	+ve	-
61	Z4B-5A	2015/10/22	41	-	-	-	-	-
62	Z4B-5B	2015/10/22	41	-	-	-	-	-
63	Z4B-5C	2015/10/22	41	-	-	-	-	-
64	U6A-5A	2015/11/02	45	30:12	-	7.90	+ve	-
65	U6A-5B	2015/11/02	45	25:12	-	6.58	+ve	-
66	U6A-5C	2015/11/02	45	30:36	-	7.43	+ve	-
67	U6B-5A	2015/11/02	45	25:48	-	6.66	+ve	-
68	U6B-5B	2015/11/02	45	37:36	-	6.56	+ve	-
69	U6B-5C	2015/11/02	45	21:18	-	9.00	+ve	-
70	Y4A-5A	2015/11/03	46	-	-	-	-	-
71	Y4A-5B	2015/11/03	46	-	-	-	-	-
72	Y4A-5C	2015/11/03	46	-	-	-	-	-
73	Y4B-5A	2015/11/03	46	-	-	-	-	-
74	Y4B-5B	2015/11/03	46	-	-	-	-	-
75	Y4B-5C	2015/11/03	46	-	-	-	-	-
76	M6A-5A	2015/11/09	42	-	-	-	-	-
77	M6A-5B	2015/11/09	42	-	-	-	-	-
78	M6A-5C	2015/11/09	42	-	-	-	-	-
79	M6B-5A	2015/11/09	42	-	-	-	-	-
80	M6B-5B	2015/11/09	42	-	-	-	-	-
81	M6B-5C	2015/11/09	42	-	-	-	-	-
82	T4A-5A	2015/11/30	46	-	-	-	-	-
83	T4A-5B	2015/11/30	46	-	-	-	-	-
84	T4A-5C	2015/11/30	46	-	-	-	-	-
85	T4B-5A	2015/11/30	46	33:12	-	6.01	+ve	-
86	T4B-5B	2015/11/30	46	27:00	-	7.86	+ve	-
87	T4B-5C	2015/11/30	46	32:00	-	6.16	+ve	-
88	U7A-5A	2015/12/20	26	-	-	-	-	-
89	U7A-5B	2015/12/20	26	-	-	-	-	-

90	U7A-5C	2015/12/20	26	-	-	-	-	-
91	U7B-5A	2015/12/20	26	-	-	-	-	-
92	U7B-5B	2015/12/20	26	-	-	-	-	-
93	U7B-5C	2015/12/20	26	-	-	-	-	-
94	Y5A-5A	2015/12/21	27	-	-	-	-	-
95	Y5A-5B	2015/12/21	27	-	-	-	-	-
96	Y5A-5C	2015/12/21	27	-	-	-	-	-
97	Y5B-5A	2015/12/21	27	-	-	-	-	-
98	Y5B-5B	2015/12/21	27	-	-	-	-	-
99	Y5B-5C	2015/12/21	27	-	-	-	-	-
100	Z5A-5A	2015/12/22	39	-	-	-	-	-
101	Z5A-5B	2015/12/22	39	-	-	-	-	-
102	Z5A-5C	2015/12/22	39	-	-	-	-	-
103	Z5B-5A	2015/12/22	39	-	-	-	-	-
104	Z5B-5B	2015/12/22	39	-	-	-	-	-
105	Z5B-5C	2015/12/22	39	-	-	-	-	-
106	U8A-5A	2016/01/04	41	-	-	-	-	-
107	U8A-5B	2016/01/04	41	-	-	-	-	-
108	U8A-5C	2016/01/04	41	-	-	-	-	-
109	U8B-5A	2016/01/04	41	-	-	-	-	-
110	U8B-5B	2016/01/04	41	-	-	-	-	-
111	U8B-5C	2016/01/04	41	-	-	-	-	-
112	Y6A-5A	2016/01/05	42	-	-	-	-	-
113	Y6A-5B	2016/01/05	42	-	-	-	-	-
114	Y6A-5C	2016/01/05	42	-	-	-	-	-
115	Y6B-5A	2016/01/05	42	-	-	-	-	-
116	Y6B-5B	2016/01/05	42	-	-	-	-	-
117	Y6B-5C	2016/01/05	42	-	-	-	-	-
118	M7A-5A	2016/01/06	33	-	-	-	-	-
119	M7A-5B	2016/01/06	33	-	-	-	-	-
120	M7A-5C	2016/01/06	33	-	-	-	-	-
121	M7B-5A	2016/01/06	33	-	-	-	-	-
122	M7B-5B	2016/01/06	33	-	-	-	-	-
123	M7B-5C	2016/01/06	33	-	-	-	-	-
124	M8A-5A	2016/01/21	48	-	-	-	-	-
125	M8A-5B	2016/01/21	48	-	-	-	-	-
126	M8A-5C	2016/01/21	48	-	-	-	-	-
127	M8B-5A	2016/01/21	48	-	-	-	-	-
128	M8B-5B	2016/01/21	48	-	-	-	-	-
129	M8B-5C	2016/01/21	48	-	-	-	-	-
130	T5A-5A	2016/02/01	35	-	-	-	-	-
131	T5A-5B	2016/02/01	35	-	-	-	-	-
132	T5A-5C	2016/02/01	35	-	-	-	-	-
133	T5B-5A	2016/02/01	35	-	-	-	-	-
134	T5B-5B	2016/02/01	35	-	-	-	-	-
135	T5B-5C	2016/02/01	35	-	-	-	-	-
136	S1A-5A	2015/06/22	33	-	-	-	-	-
137	S1A-5B	2015/06/22	33	19:42	-	7.18	+ve	-
138	S1A-5C	2015/06/22	33	-	-	-	-	-

139	S1B-5A	2015/06/22	33	-	-	-	-	-
140	S1B-5B	2015/06/22	33	16:30	-	8.30	+ve	-
141	S1B-5C	2015/06/22	33	18:00	-	7.76	+ve	-
142	Y1A-5A	2015/07/08	33	19:18	-	7.40	+ve	-
143	Y1A-5B	2015/07/08	33	24:12	-	5.65	+ve	-
144	Y1A-5C	2015/07/08	33	18:12	-	7.43	+ve	-
145	Y1B-5A	2015/07/08	33	18:06	-	7.43	+ve	-
146	Y1B-5B	2015/07/08	33	20:18	-	5.54	+ve	-
147	Y1B-5C	2015/07/08	33	27:48	-	5.26	+ve	-
148	T6A-5A	2016/02/10	44	-	-	-	-	-
149	T6A-5B	2016/02/10	44	-	-	-	-	-
150	T6A-5C	2016/02/10	44	-	-	-	-	-
151	T6B-5A	2016/02/10	44	-	-	-	-	-
152	T6B-5B	2016/02/10	44	-	-	-	-	-
153	T6B-5C	2016/02/10	44	-	-	-	-	-
154	Z6A-5A	2016/02/15	34	-	-	-	-	-
155	Z6A-5B	2016/02/15	34	-	-	-	-	-
156	Z6A-5C	2016/02/15	34	-	-	-	-	-
157	Z6B-5A	2016/02/15	34	-	-	-	-	-
158	Z6B-5B	2016/02/15	34	-	-	-	-	-
159	Z6B-5C	2016/02/15	34	-	-	-	-	-
160	Z7A-5A	2016/02/29	48	-	-	-	-	-
161	Z7A-5B	2016/02/29	48	-	-	-	-	-
162	Z7A-5C	2016/02/29	48	-	-	-	-	-
163	Z7B-5A	2016/02/29	48	-	-	-	-	-
164	Z7B-5B	2016/02/29	48	-	-	-	-	-
165	Z7B-5C	2016/02/29	48	-	-	-	-	-

* Of the eight samples, all were shown to be negative by direct plating, but five were positive by enrichment culturing.

(-), negative; no *C. jejuni* or *C. coli* detected by LAMP or by isolation.

(+ve), *Campylobacter* successfully isolated.
